# Structural Dynamics of Lys11-Selective Deubiquitinylase Cezanne-1 during the Catalytic Cycle

**DOI:** 10.1021/acs.jcim.2c01281

**Published:** 2023-03-21

**Authors:** Metehan Ilter, Eric Schulze-Niemand, Michael Naumann, Matthias Stein

**Affiliations:** † Molecular Simulations and Design Group, Max Planck Institute for Dynamics of Complex Technical Systems, 39106 Magdeburg, Germany; ‡ Medical Faculty, Institute for Experimental Internal Medicine, Otto von Guericke University, 39120 Magdeburg, Germany

## Abstract

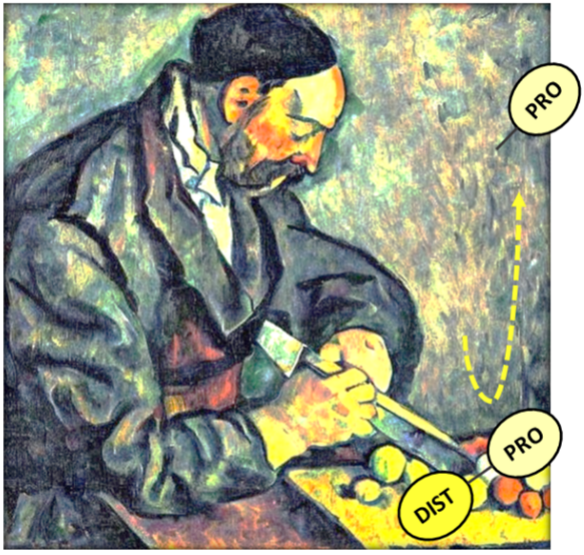

Deubiquitinylating enzymes (DUBs) regulate the deubiquitinylation process of post-translationally modified proteins and thus control protein signaling in various cellular processes. The DUB Cezanne-1 catalyzes the cleavage of the iso-peptide bond of Lys11-linked polyubiquitin chains with high selectivity. Crystal structures of Cezanne-1 in different states provide important insight regarding the complex formation and global changes during the catalytic cycle but are lacking details of dynamics and control of activation. Activity-based probes are used to isolate intermediate states upon forming covalent bonds with the DUB active site. Those, however, may lead to structures that are non-native. Conformational changes of Cezanne-1, during its process of activation and proteolytic activity, are investigated using all-atom molecular dynamics (MD) simulations of the ubiquitin-free, diubiquitin-bound, and monoubiquitin-bound Cezanne-1 DUB for a total of ∼18 μs. Our results show that ubiquitin-free Cezanne-1 dynamically shuttles between catalytically competent and incompetent states which suggests that its activation is independent of substrate binding. The catalytically competent substrate-free Cezanne-1 promotes distal ubiquitin substrate access to the catalytic center. The subsequent binding of the proximal ubiquitin shifts the equilibrium toward the catalytically competent state of the dyad, thereby promoting proteolysis of the iso-peptide bond. After cleavage of the scissile bond, sequential dissociation of first the proximal ubiquitin induces the inactivation of Cezanne-1. The subsequent release of the distal ubiquitin fully reconstitutes the inactive substrate-free state of Cezanne-1. The process of activation and catalytic turnover of DUB Cezanne-1 is a multistage cycle with several critical dynamic transitions that cannot be characterized based on protein structures alone. Activity-based probes of cysteine proteases lead to non-native protein–protein contacts, which need to be resolved in order to be able to issue statements about physiological states and substrate binding.

## Introduction

Ubiquitinylation, i.e., the covalent attachment of one or several ubiquitin moieties to target proteins, is an important post-translational modification (PTM) and a signal for proteasomal degradation.^1,2^ The PTM is carried out by an enzymatic cascade of ubiquitin-activating enzymes (E1), ubiquitin-conjugating enzymes (E2), and ubiquitin-ligating enzymes (E3).^3^ Ubiquitin is covalently conjugated to target proteins via the formation of an iso-peptide bond between the C-terminus of the ubiquitin and the ϵ-amino group of a lysine residue of the target protein (or the N-terminus of the target protein).^4^

Besides monoubiquitinylation, polyubiquitinylation, i.e., successively conjugating several ubiquitin units, is more frequent.^5^ In longer polyubiquitin chains, the variation in the linkage between the different ubiquitin monomers via lysine amino acid residues (K6, K11, K29, K33, K48, and K63) or methionine (M1) allows selective regulatory control of cellular processes, such as protein degradation, cell signaling, and cell cycle regulation.^6^

Enzymatic cleavage and removal of ubiquitin tags from proteins are performed by deubiquitinylases (DUBs), some of which selectively recognize the type of lysine/methionine linkage and initiate downstream signaling.^7^ Hence, the dysregulation of DUB activity in cellular processes can contribute to tumorgenesis and other diseases.^8^

So far, a total of ∼100 human DUBs have been identified which are categorized into seven subfamilies: (i) ovarian tumor domain (OTU)-containing proteases, (ii) ubiquitin C-terminal hydrolases (UCHs), (iii) ubiquitin-specific proteases (USPs), (iv) Machado-Joseph domain papain-like proteases (MJDs), (v) motif interacting with ubiquitin-containing novel DUB family (MINDY), (vi) zinc-finger-containing ubiquitin-specific protease (ZnF-UBP), and (vii) JAB1/MPN/MOV34 metalloenzymes (JAMMs).^7,9^

In addition to eukaryotic DUBs, many bacterial species express and release DUBs in the host cell.^100^ These proteins are acquired from the host genomes in several independent processes, but are only distantly related to eukaryotic DUBs and are not easily assignable to any of the seven eukaryotic DUB families.^10^

The purpose of bacterial DUBs is to reverse the ubiquitinylation and prevent pathogen proteins from degradation. The ubiquitin receptors (p62 and optineurin) recognize K63- or M1-chains within the bacteria-containing vacuoles.^11^ Thus, most bacterial DUBs are either K63 specific or absolutely nonselective with the exception of RavD, which specifically cleaves M1-chains.^12,13^ Interestingly, some bacterial DUBs not only target ubiquitin but also ubiquitin modifiers that are encoded by the host.^10^ Moreover, there are several virus-encoded deubiquitinylases that significantly affect viral infection.^14^

Among the DUBs in human, the OTU subfamily is distinct from other DUBs by its selective recognition and cleavage of certain specific ubiquitin linkages.^15^ The 16 members of the human OTU family display a range of diubiquitin cleavage preferences for K6, K11, K48, K63, and M1;^9^ by contrast, USP family members can hydrolyze all linkages and display low linkage selectivity.^16^

The OTU family member Cezanne-1 (OTUD7B) was identified as the first OTU with a high selectivity toward K11-linked polyubiquitin chains^17^ and performs its proteolytic activity by a cysteine/histidine catalytic dyad. Substrate specificity of Cezanne-1 is mediated by a glutamate residue Glu157 in the vicinity of the dyad, which is critical for enzyme activity but involved in bond cleavage.^18^

The central role of K11-linked polyubiquitins in the mammalian cell cycle has generated a new interest in K11-selective ubiquitinylating enzymes and DUBs.^19−22^ Cezanne-1 is involved in nuclear factor-κ B (NF-κB) signaling, T-cell activation, and homeostasis of hypoxia-inducible factor 1-alpha (HIF-1α) and 2-alpha (HIF-2α).^23−28^ The overexpression of Cezanne-1 correlates with short survival of both breast and nonsmall cell lung cancer patients.^29,30^ Cezanne-2 (OTUD7A) has the same selectivity toward K11-linked polyubiquitin chains^18,31^ but is not significantly overexpressed in cancer cells.^29^ Thus, Cezanne-1 is an attractive target in the development of therapeutics against cancer.^32^

OTU Cezanne-1 contains three domains: the catalytic OTU domain, the ubiquitin-associated domain (UBA), and an A20-like zinc finger domain (ZnF), of which the latter two belong to the ubiquitin-binding domains (UBDs). The OTU domain performs the deubiquitinylase activity.^18^

Covalent modifiers to the cysteine protease were necessary to enable the crystallization in different, otherwise elusive states. Activity-based probes (APBs) are commonly used to stabilize substrate-bound or product states of DUBs by forming covalent bonds to the nucleophile cysteine sulfur atom using ‘click chemistry’.^33,34^ Those covalent modifiers may introduce alterations of contacts in the protein–protein crystal structures that do not reflect the physiological states.

Protein crystal structures of the OTU domain of Cezanne-1 were obtained for different states of the DUB during its catalytic process. The substrate-free, diubiquitin substrate bound and the product monoubiquitinylated structures give critical insight into global structural changes during the catalytic turnover. The substrate-bound and product states were crystallized in the presence of ABPs in order to be able to covalently trap these intermediates. The resolved crystal structures point to the molecular basis of the K11-linked polyubiquitin specificity of Cezanne-1 and a ubiquitin-assisted activation process.^18^ However, the static crystal structures do not allow statements regarding physiologically relevant conformational flexibility and local conformational changes of Cezanne-1 during its catalytic turnover.

In the absence of substrate, Cezanne-1 was crystallized in a catalytically incompetent autoinhibited state, in which a nearby Cys-loop obstructs substrate access and the process of activation of the catalytic dyad. Hydrogen–deuterium exchange mass spectrometry (HDX-MS), however, points to the dynamic nature of Cezanne-1 in solution and thus the presence of one or several substrate-free conformational states.^18^ In the crystallized state, substrate access would not be possible due to a strong Leu294–Asn193 interaction.

When the substrate-bound Cezanne-1 was crystallized with an ABP-modified substrate mimic, its dyad residue was suggested to be in an inactive ‘His358-out’ conformation to afford the substrate residue Lys33 approaching the catalytic site residue Glu157 of Cezanne-1.

Sequence modifications of Cezanne-1 in the long, unstructured V-loop (residues 267–291) by a truncated three-residue fragment (Gln-Pro-Gly) of TRABID were necessary when crystallizing the monoubiquitinylated Cezanne-1 structure. The monoubiquitinylated Cezanne-1 crystal structure contains two distinct complexes in the asymmetric unit. One complex has a catalytically competent dyad, whereas the second reveals a catalytically noncompetent dyad. Also, in the complex with the competent catalytic dyad, both ‘His-in’ and ‘His-out’ conformations are present with occupancies of 0.53 and 0.47, respectively. Thus, the suggested transition from an inactive to an active state of Cezanne-1 is unlikely. It is, according to our findings, rather the opposite deactivation step in Ub-Cez (from active to inactive) that completes the catalytic cycle.

Molecular dynamics (MD) simulations are able to sample protein conformational changes, intermediates, and transient states at an atomic spatiotemporal resolution.^35^ Prior to the simulations, the native DUB structures can be reconstituted by removing covalent modifiers and mutations that were necessary to crystallize the protein in different states.

Previous MD simulations of human OTU proteins OTUB1, OTUB2,^36^ OTULIN, and its functional bacterial analogue RavD^13^ have provided insight that could not be obtained from crystal structures alone. Solvent accessibility of the active center, substrate recognition via ubiquitin binding sites S1 and S1′, and substrate-induced conformational changes were revealed. It could also be shown that modifications of the diubiquitin lead to a substrate-bound structure that does not correspond to that of physiological substrate recognition.^37^

Our MD simulations, covering a total of 18.3 μs for Cezanne-1 in different states along the deubiquitinylating process, reveal the dynamics of conformational changes of this OTU during its enzymatic performance. The MD simulations show that substrate binding is not necessary for the activation of Cezanne-1. Even in absence of diubiquitin, there is an equilibrium between the inactive and active states of the catalytic dyad. Substrate binding, however, shifts the equilibrium toward the catalytically active state by means of an electrostatic stabilization of the substrate by a glutamate residue. Although this residue is not actively involved in iso-peptide bond cleavage, it is catalytically relevant in terms of correct substrate positioning close to the catalytic center. The release of the cleaved ubiquitin monomers is sequential and initiated by dissociation of the proximal ubiquitin, reconstitution of the inactive state of Cezanne-1, and then release of the distal ubiquitin. This information cannot be obtained from protein crystallographic studies alone but is important when exploring Cezanne-1 as a drug target and aiming at selectively inhibiting a particular state of function.

## Methods

### Structural Details

Protein crystal structures of the OTU domain of human Cezanne-1 were retrieved from the Protein Data Bank.^38,39^ The obtained structures were ubiquitin-free (*Cez*_*apo*_) (PDB ID: 5LRU), diubiquitin-bound (*CezUb*_2_) (PDB ID: 5LRV), and the active state of monoubiquitin-bound form (*Cez*_*act*_*Ub*_*DIST*_) (PDB ID: 5LRW-chain A).^18^

Mutations in the protein structures of Cezanne-1 (Met128 and Lys439) were changed back to those of wild-type Cezanne-1 (Leu and Asp). The V-loop of the monoubiquitin-bound Cezanne-1 (residues 267–291) was recovered by substituting the introduced TRABID sequence (Gln-Pro-Gly; QPG) in the crystal structure with the native Cezanne-1 sequence. For *Cez*_*act*_*Ub*_*DIST*_, the ubiquitin-based suicide probe in the crystal structure was removed to recover the physiological distal ubiquitin binding to Cezanne1.

The covalent activity-based probe, which allowed the crystallization of *CezUb*_2_, was removed, and the native distal and proximal ubiquitin connectivities were reconstructed. The free active site cysteine residue was recovered.

Missing regions in the crystal structures were remodeled using the DOPE-HR loop-modeling protocol of MODELER in UCSF Chimera.^40,41^ Residue protonation at pH 7.4 was performed with the Protein Preparation Wizard.^42^ The protonation states of the catalytic residues Cys194 and His358 were assigned to correspond to neutral (Cys194^0^/His358^0^) and zwitterionic (Cys194^–^/His358^+^) charge states for apo Cezanne-1. For di- and monoubiquitinylated Cezanne-1, the zwitterionic state, which corresponds to an active catalytic site, was simulated.

Visual Molecular Dynamics (VMD) was used to set up the simulation boxes.^43^ The systems were solvated with TIP3P water molecules and neutralized at 0.15 M of NaCl.^44^ The CHARMM36m force field was used in all simulations.^45^

### Details of Molecular Dynamics Simulations

All simulations were performed using OpenMM.^46^ The nonbonded interactions were calculated with a cutoff of 1.2 nm and a switch distance of 1.0 nm. The long-range electrostatic interactions were calculated by making use of particle mesh Ewald (PME) summation with an error tolerance of 0.00001.^47,48^ The Langevin integrator with a 1 ps^–1^ friction coefficient and 2 fs time step was employed to keep the temperature constant at 310 K.^49^ The pressure of 1 bar was controlled by a Monte Carlo barostat, which was coupled every 25 integration steps.^50^ All MD simulations were performed at pH 7.4. The starting structures were minimized for 1000 steps. Each state of Cezanne-1, namely, Cez^0^_*apo*_, *Cez^+/-^*_*apo*_, *Cez^+/-^Ub*_2_, and *Cez^+/-^*_*act*_*Ub*_*DIST*_, was simulated in triplicates of ca. 1 μs production period (see Table 1). Different initial velocities were assigned according to Maxwell distributions in the equilibration step. A total simulation time of ca. 18 μs was reached.

**Table 1:** Overview of Performed MD Simulations

Simulated state of Cezanne-1	Protonation state of the catalytic dyad	Abbreviation	Number of replicates	Total simulation time (μ**s)**
ubiquitin-free	neutral	*Cez*^0^_*apo*_	3	3.31
ubiquitin-free	zwitterionic	*Cez*^+/-^_*apo*_	3	3
diubiquitin-bound	zwitterionic	*Cez*^+/-^*Ub*_2_	8	6.03
monoubiuqitin-bound	zwitterionic	*Cez*^+/-^_act_*Ub*_*DIST*_	6	6

In addition to simulations starting from crystal structures, we also initiated simulations from selected MD snapshots to enhance the sampling of states. In this way, the bias of starting the simulations from covalently inhibited nonphysiological structures was circumvented. To enrich the conformational sampling of the active substrate-bound *Cez*^+/-^*Ub*_2_ state, an MD snapshot with an inter-residue distance less than 0.4 nm for the dyad was picked, which represents the active state of *Cez^+/-^Ub*_2_. The system was simulated in five replicates for a total of 3 μs.

In order to sample the dissociation of the proximal ubiquitin from *Cez*^+/-^*Ub*_2_, a snapshot of an active state and an estimated high K_d_ of the proximal ubiquitin was selected (see below). The proximal ubiquitin was then manually removed. Thus, structural relaxations during the transition from diubiquitin- to monoubiquitin-bound Cezanne-1 could be sampled. The system was simulated three times for 1 μs (3 μs total simulation time).

### Calculation of Ubiquitin Dissociation Constants

The dissociation constants (K_d_) of the PRO and DIST ubiquitins from *Cez*^+/-^*Ub*_2_ were calculated for a set of 3000 equidistant MD frames at an interval of 1 ns from trajectories of substrate-bound, active Cezanne-1. K_d_ values of PRO and DIST ubiquitin were obtained at 310.15 K with PRODIGY.^51,52^ PRODIGY makes use of an analysis of the number of interfacial contacts of protein–protein complexes which were correlated with experimental binding affinities.

### Analysis of Trajectories

The MD trajectories were analyzed using tools from GROMACS.^53,54^ Data visualization was carried out by making use of the Seaborn and Matplotlib libraries.^55,56^ The trajectories were visualized and rendered with VMD and Tachyon ray tracing.^43,57^

The root-mean-square deviation (RMSD) of backbone atoms of the PRO and DIST ubiquitins from their orientations in the crystal structure were calculated by aligning the trajectories to the OTU domain of the *CezUb*_2_ crystal structure.

The numbers of contacts of PRO and DIST ubiquitin residues with the Cezanne-1 binding sites were calculated using a minimum contact distance cutoff of 0.55 nm. The average numbers of contacts of PRO and DIST ubiquitins are shown as a bar plot along with the standard error of the mean in Figure 6B.

The interatomic distances between (i) Sγ-Cys194 and Nδ1-His358 (Cys194···His358), (ii) Oϵ1-Glu157 and Nϵ2-His358 (Glu157···His358), and (iii) Sγ-Cys194 and C-Gly76 of DIST ubiquitin (Cys194···Gly76), and (iv) minimum distances between Oϵ1/Oϵ2-Glu157 of Cezanne-1 and Nζ-Lys33 of PRO ubiquitin (Glu157···Lys33) were used in free energy contour maps. The 2D joint probability density functions of interatomic distances were first estimated by dividing the timeline data into 50 × 50 bins using the NumPy library.^58^ The negative natural logarithm of the probability densities was multiplied by Boltzmann’s constant and temperature. The calculated free energy values were normalized.

Principal component analysis (PCA) of atomic motion was used to reveal differences in the collective dynamics between ubiquitin-free and diubiquitin-bound Cezanne-1. The covariance matrix of Cα atoms was generated, and trajectories were projected along the first three eigenvectors, which accounted for at least ca. 40% of the overall dynamics (see left and right panels in Figure S1A). Then, the Cα RMSFs of the projected *Cez*^+/-^_*apo*_ and *Cez*^+/-^*Ub*_2_ trajectories were computed.

## Results and Discussion

### Catalytic Dyad of Substrate-Free Cezanne-1 Is Shuttling between Competent and Incompetent States

The formation of a charge-separate state is a prerequisite for the catalytic activity of cysteine proteases. Orientation and inter-residue distances of catalytic residues are thus indicators of the prevalence of a catalytically competent or noncompetent state of a DUB.^13,36,37,59,60^

**Figure 1:**
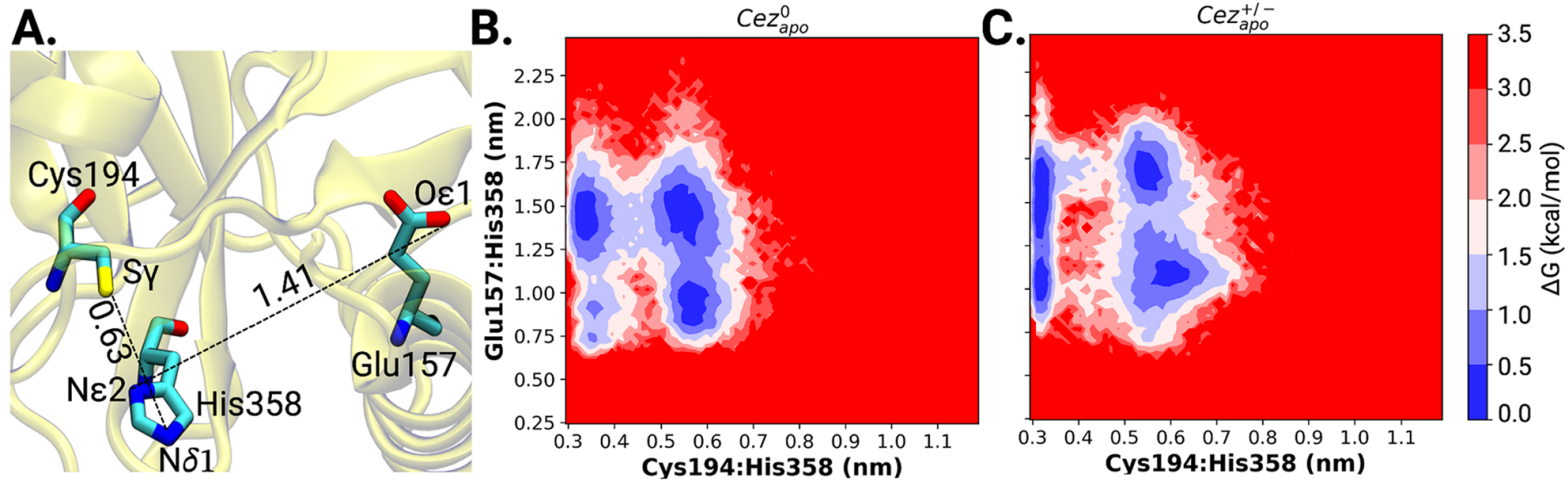
Inter-residue distances of the active site from the crystal structure and MD sampling. (A) Representation of the catalytic center of substrate-free Cezanne-1 (crystal structure PDB ID: 5LRU). Critical inter-residue distances (Cys194···His358 and Glu157···His358) are labeled. Free energy contour maps of Cys194···His358 and Glu157···His358 inter-residue distances for (B) neutral (*Cez*^0^_*apo*_) and (C) charge separate (*Cez*^+/-^_*apo*_) states of substrate-free Cezanne-1. Cys194···His358 inter-residue distances of 0.4 nm and below are indicative of a catalytically competent state.

Inter-residue distances between the catalytic dyad residues Cys194···His358 and His358···Glu157 (Figure 1.A) are used as structural identifiers to characterize the state of catalytic competency of the active site of Cezanne-1. In the crystal structure of substrate-free Cezanne-1 (PDB ID: 5LRU), the Cys194···His358 distance is 0.63 nm and thus indicative of a catalytically noncompetent state. At a long distance of 1.41 nm between Glu157 and His358 promotion of the catalytic dyad orientation and ionization appears not feasible.

The MD simulations reveal, however, the dynamic behavior of the catalytic dyad residues in absence of a diubiquitin substrate (Figure 1B and C).

The free energy contour maps of the inter-residue distances show two protonation state-independent energy wells of similar depth at Glu157···His358 distances of 0.75 and 1.75 nm. The energy barrier between the two states is only around 0.5–1.0 kcal/mol, which enables rapid transitions between the minima. This is also seen in the MD trajectories (see the top panel in Figure S2). Since the sampling of Glu157···His358 is not affected by the state of activation of Cezanne-1, the involvement of Glu157 in the reaction needs to be investigated in detail.

The energy minima of the Cys194···His358 distances are occurring at 0.35 and 0.55 nm for *Cez*^0^_*apo*_ and at 0.31 and 0.57 nm for *Cez*^+/-^_*apo*_ (Figure 1B and C). The free energy barriers between the states are 1.5 kcal/mol (*Cez*^0^_*apo*_) and 2 kcal/mol (*Cez*^+/-^_*apo*_), and several transitions can be observed within 3 μs of cumulative MD sampling (see the bottom panel in Figure S2). It can be seen that the close positioning and correct orientation of His358 with respect to Cys194 occurs independently of the Glu157···His358 interaction. Apparently, residue Glu157 does not directly promote the formation of a catalytically competent active site. Considering a Cys194-His358 distance of 0.4 nm and below as indicative of catalytic competency, the probability to find substrate-free Cezanne-1 in a catalytically competent low energy state is 0.25 in a neutral state and 0.51 in a zwitterionic charge state. Thus, substrate-free Cezanne-1 is more prone to adopt a catalytically noncompetent conformation. After charge separation of the catalytic residues in a non-competent conformation, the probability of formation of a competent state is higher, albeit both states of activation are occurring with almost equal probability.

### Substrate Accessibility Is Linked to the Formation of the Zwitterionic State of the Dyad

**Figure 2:**
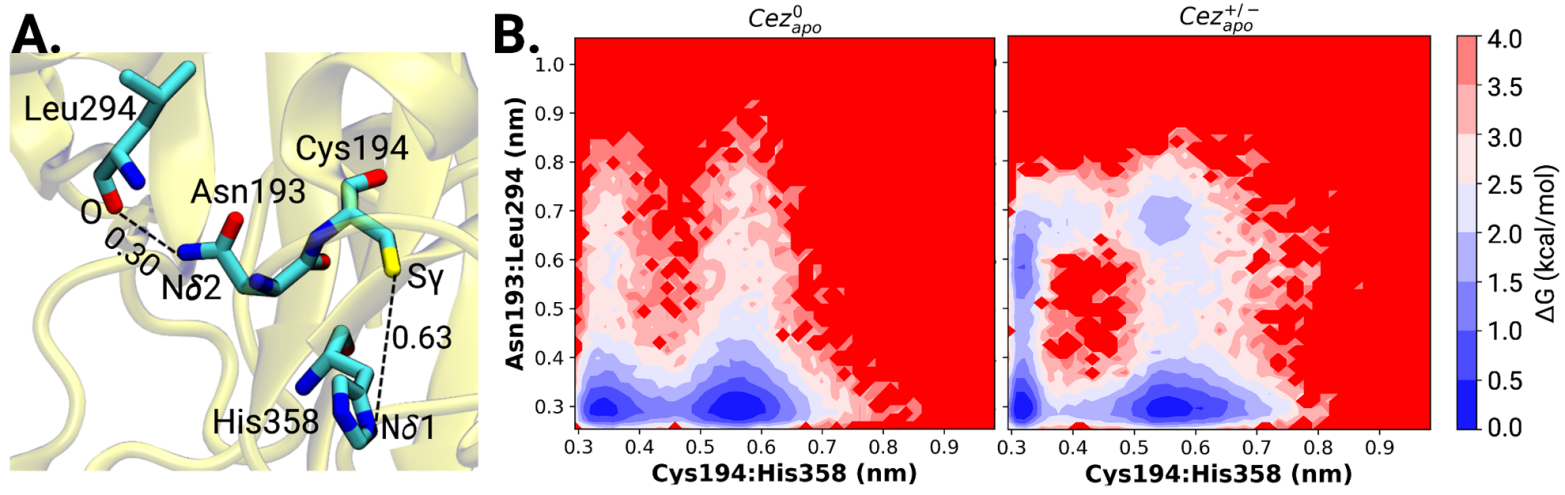
Zwitterionic charge state of the catalytic dyad enables Ub access. (A) Orientation and inter-residue distances of Cys194···His358 and Asn193···Leu294 in the autoinhibited Cez_apo_ crystal structure (PDB ID: 5LRU). (B) Free energy contour maps of Cys194···His358 and Asn193···Leu294 distances in neutral (*Cez*^0^_*apo*_) and charge separate (*Cez*^+/-^_*apo*_) states. Free energy maps are calculated from 3 μs of cumulative MD simulation.

Substrate-free Cezanne-1 is autoinhibited by sterically hindered substrate access toward the catalytic center. Such blockage is due to an interaction between residues of the Cys-loop residue Asn193 and Leu294 as indicated by a short distance (0.3 nm) in the crystal structure (Figure 2A).

In order to gain insight into the control of substrate access by the Cys-loop residues, Cys194···His358 and Leu294···Asn193 distances were monitored and plotted in a free energy map (Figure 2B). In particular, for the uncharged catalytic dyad, short Leu294···Asn193 distances below 0.4 nm are predominant. After proton transfer, further energy minima at longer Leu294···Asn193 distances from 0.55 to 0.70 nm appear, which correspond to an open Cys-loop orientation. The zwitterionic charge state of the dyad thus promotes the catalytic competency of Cezanne-1 by lifting the autoinhibition of the crystallized state. Only the charge-separated state of the catalytic dyad is thus considered further.

It can be shown that, upon formation of the zwitterionic state of apo Cezanne-1, the hydrogen bond interaction between the backbone amide of Cys194 and side-chain oxygen of Asn193 (Figure S3A) is removed, which leads to the increase in the Asn193···Leu294 distance (from 0.3 to 0.68 nm; see Figure S3C). Thus, the formation of the charge-separate active state in substrate-free Cezanne-1 leads to an increase in Asn193···Leu294 distances which then subsequently enables the C-terminus approach of the distal ubiquitin to the catalytic site of Cezanne-1.

### Investigation of Substrate-Bound Cezanne-1 Dynamics upon Reconstitution of the Physiological Binding Mode

**Figure 3:**
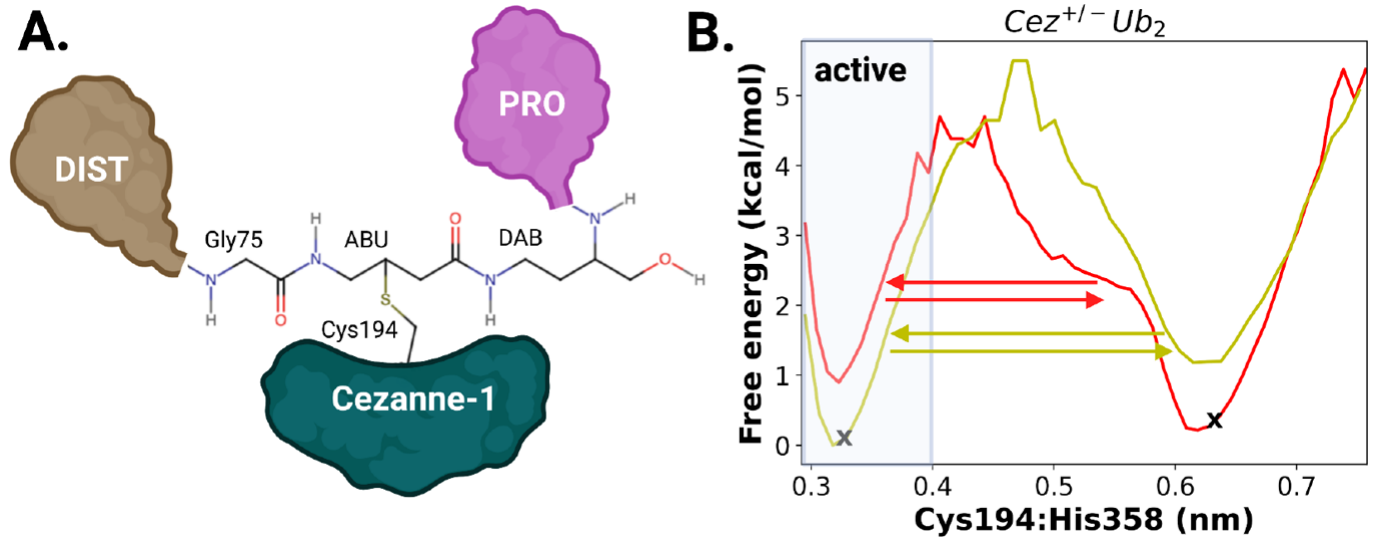
Initial configuration bias in the ABP co-crystallized diubiquitin Cezanne-1 complex. (A) Cartoon of the ABP-trapped diubiquitin substrate-bound Cezanne-1 complex. The native iso-peptide bond between Gly76-DIST and Lys11-PRO residues is substituted by gamma-amino-butanoic acid (ABU) and 2,4-diaminobutyric acid (DAB), which form a covalent bond with the proteolytic Cys194 residue. (B) Free energy profiles as a function of the Cys194···His358. Simulations of the red curve started from the reconstituted native DIST-Gly75-Gly76-Lys11-PRO Cezanne-1 diubiquitin structure (from PDB ID: 5LRV). The initial Cys194···His358 distance was 0.63 nm as denoted by a cross. Simulations along the yellow curve initiated from a diubiquitin bound Cezanne-1 in a catalytically competent state with short Cys194···His358 distance (0.32 nm).

Activity-based protein profiling (ABPP) has emerged as a powerful strategy to label and identify elusive protein intermediates. Highly selective active-site targeting chemical probes are used to trap those states *in situ* by forming covalent bonds between the ABP and the protein. For Cezanne-1, the synthetic diubiquitin construct contains an electrophile after the distal ubiquitin Gly75 residue and before the proximal ubiquitin residue Gly10.^18,61,62^ The ABP uses a synthetic linker consisting of gamma-amino-butanoic acid (ABU) and 2,4-diaminobutyric acid (DAB) to replace distal ubiquitin residue Gly76 and proximal ubiquitin residue Lys11 (Figure 3A). The electrophile probe forms a covalent bond with the protease cysteine sulfur atom.

**Figure 4:**
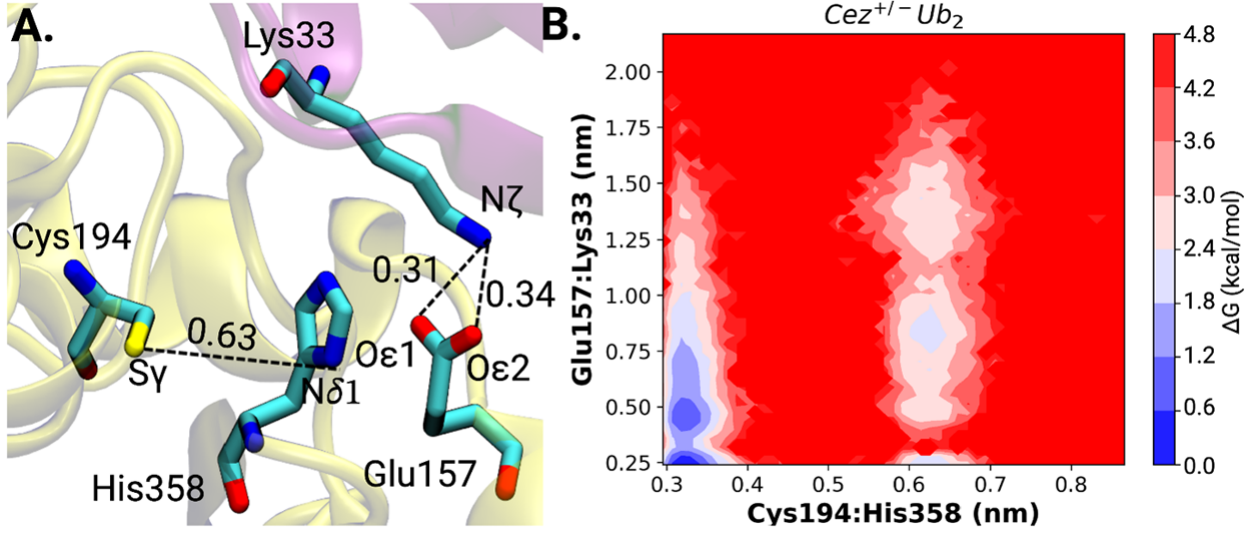
Structure and dynamics of K11-linked diubiquitin recognition by Cezanne-1. (A) Structural details of diubiquitin bound Cezanne-1 modified by an ABP (PDB ID: 5LRV). Cezanne-1 residues Glu157, Cys194, and His358, as well as that of the proximal ubiquitin substrate Lys33 are labeled. The OTU domain of Cezanne-1 is in yellow, and PRO ubiquitin is in purple. The large inter-residue distances are indicative of a catalytically inactive substrate-bound Cezanne-1 complex. (B) Free energy contour map of di-Ub Cys194···His358 and Glu157···Lys33 distances for physiologically reconstituted di-Ub. The distances were sampled from five independent replicas of MD simulations which were initiated from a catalytically competent conformation.

In the substrate-bound crystal structure of Cezanne-1 (PDB ID: 5LRV), a covalent bond is formed between the Cβ atom of the synthetic linker and the Sγ atom of the catalytic Cys194 (at a usual distance of a C–S covalent bond of 0.18 nm). Although the cocrystal aims at trapping a substrate-bound Cezanne-1 *in situ* during turnover, the Cys194···His358 distance of 0.63 nm (Figure 4A) is clearly indicative of a catalytically noncompetent state, which is inactive and cannot perform cleavage of the scissile bond. To investigate the native diubiquitin Cezanne-1 complex, the physiological DIST-Gly75-Gly76-Lys11-PRO iso-peptide linkage was reconstituted. MD simulations from the reconstituted crystal structure (Cys194···His358 = 0.63 nm) yield a bimodal distribution of Cys194···His358 distances with clear energy wells at 0.33 and 0.62 nm (Figure 3B). The free energy difference from incompetent (0.62 nm) to competent (0.33 nm) states is estimated to be −1 kcal/mol with an energy barrier of ∼4.5 kcal/mol. However, starting MD simulations from a conformation with short Cys194···His358 distances of 0.4 nm and below, the competent state is favored. The absolute free energy difference remains similar, and the energy barrier increases to 5.5 kcal/mol. The initial configuration bias indicates the difficulty of MD simulations to sample high-energy transition states sufficiently.

It can, however, be concluded that the covalent ABP leads to the crystallization of Cezanne-1 in a nonphysiological, here, catalytically inactive state. Upon binding of the substrate, Cezanne-1 must adopt a catalytically active state in order to perform the scissile iso-peptide bond cleavage. Such a prereactive state was sampled, and a representative snapshot is depicted in Figure 7A.

### Recognition of K11-Linked Proximal Ubiquitin Boosts Catalytic Competency

Cezanne-1 is a highly selective DUB toward K11-linked ubiquitin chains. It has been shown that residue Glu157 is critical for the K11 selectivity and catalytic turnover.^18^ When replaced by another amino acid residue, substrate binding affinity K_M_ and k_cat_ are affected (see below). In the complex with an ABP-trapped diubiquitin analogue (PDB ID: 5LRV), the proximal ubiquitin residue Lys33 is in close contact with Glu157 of Cezanne-1 (Oϵ1:Nζ and Oϵ2:Nζ Glu157···Lys33 distances of 0.31 and 0.34 nm), albeit with the active site being catalytically inactive (Figure 1A). In the crystal structure, formation of the close contact between Glu157 (Cezanne-1) and Lys33 (PRO) is only possible in the ‘His358-out’ conformation.^18^

In the substrate-free state, the catalytic dyad of Cezanne-1 dynamically shuffles between two equally probable conformations, one of which is catalytically competent (see above). In the reconstituted native complex of K11-linked diubiquitin and Cezanne-1, the catalytically competent state is favored by 2.4 kcal/mol when interactions between Glu157 (Cezanne-1) and Lys33 (PRO) are occurring below 0.5 nm (Figure 4B) due to electrostatic stabilization of the ion pair. Thus, substrate binding stabilizes the active state of the catalytic dyad prior to performing its enzymatic function. This information is not available from the X-ray structure of ABP-modified *CezUb*_2_, which crystallized in an inactive state. The strong electrostatic interaction is persistent for 60% of the total simulation time. The role of Glu157 for the selective recognition and proteolysis of K11-linked diubiquitin is also apparent from mutations in enzyme and substrate. The Glu157Ala Cezanne-1 mutant showed a reduced and slower proteolytic activity. The charge-inverting Glu157Lys Cezanne-1 mutant showed an even further reduced activity and loss of selectivity. When mutations in diubiquitin substrate were introduced, Lys33Ala or Lys33Glu only showed diminished cleavage when in complex with wild-type Cezanne-1.^18^ Residue Glu157 is thus critically relevant for enzymatic catalysis, although it may not be directly involved in cleaving the iso-peptide bond. This is in agreement with our results of a strong enzyme–substrate interaction between Glu157 and Lys33 in a catalytically competent dyad state.

### Substrate-Induced Conformational Changes of Cezanne-1

A comparison of crystal structures of substrate-free and substrate-bound Cezanne-1 reveals no large-scale conformational changes upon substrate binding. However, some minor local conformational changes can be observed within the ubiquitin-binding sites. In the proximal binding site S1′, helix α1 (residues 146–153) undergoes a 90° rotation, and helix α2 (residues 158–168) shortens by 1.5 windings. The loop between helices α3 and α4 (residues 199–208) is positioned toward helix α1.^18^ Additionally, the long flexible loop (V-loop) located between the two ubiquitin-binding sites is not fully resolved in the crystal structures.

**Figure 5:**
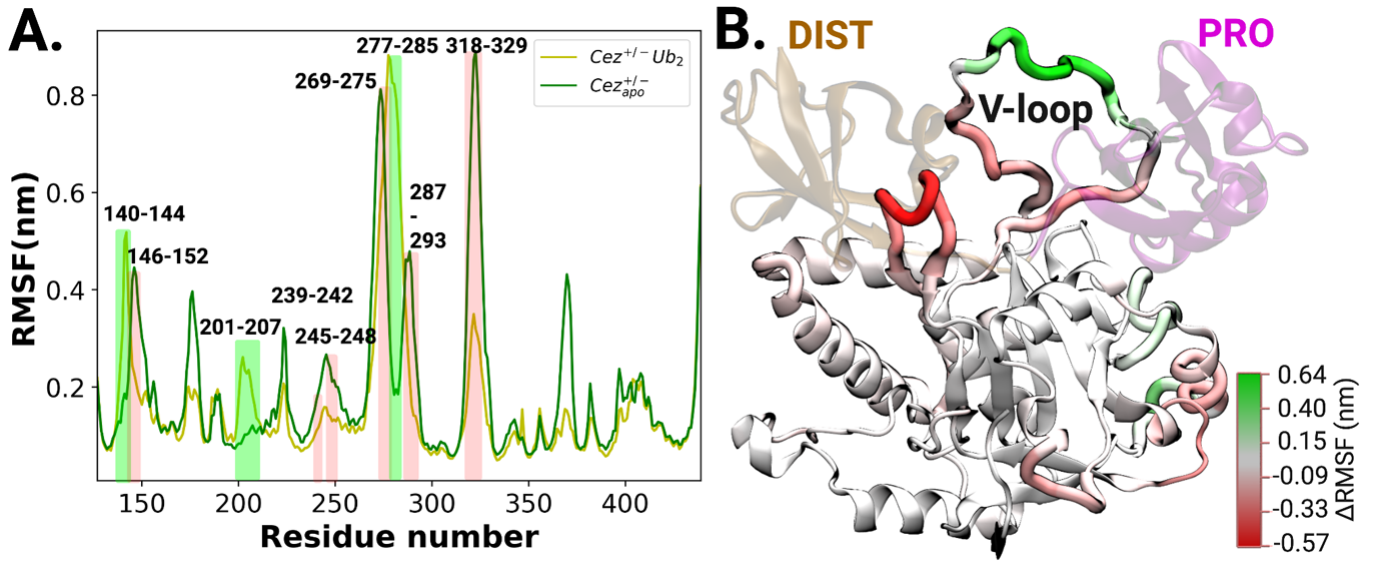
Changes in residual fluctuations of Cezanne-1 upon diubiquitin binding. (A) Comparison of backbone root-mean-square fluctuations (RMSF) of Cezanne-1 residues in the absence and presence of diubiquitin substrate. Residues in the proximity of PRO and DIST ubiquitin-binding sites are explicitly labeled. Stretches of residues with an increase in flexibility are highlighted green, and residues that are stabilized upon di-Ub binding are highlighted red. (B) Structural mapping of changes in RMSF of Cezanne-1 residues (PDB ID: 5LRV). PRO and DIST ubiquitins are shown in purple and ochre, respectively. Residues with changes in backbone RMSF of ≥ ± 0.1 nm are colored from red to green.

Local conformational changes upon substrate binding can be identified by inspection of changes in root-mean-squared fluctuations (ΔRMSFs) (Figure 5). Residues of the distal ubiquitin-binding site or in proximity, e.g., 239–242, 245–248, and 318–329, display a significant reduction in RMSFs upon substrate binding. Residues 140–144 and 201–207, however, which belong to the proximal ubiquitin-binding site display an increase in flexibility. Residues 146–152 of helix α1 are structurally stabilized in the substrate-bound Cezanne-1 complex.

The V-loop comprising residues 267–291 is structurally unresolved. The modeled V-loop can form an interaction interface with both proximal and distal ubiquitins. Upon substrate binding, V-loop residues 269–275 and 287–291 become markedly more stable, whereas residues 277–285 become more flexible. These flexibility changes within the V-loop are further supported by principal component analysis (PCA), in which the V-loop of residues from 275 to 286 are identified as major contributors to the conformational variance of Cezanne-1 (see Figure S1B).

Overall, substrate binding stabilizes the distal ubiquitin-binding site and destabilizes the proximal binding site and the V-loop of Cezanne-1. This is also supported by the changes in Cα RMSD per residue in comparison to the substrate-bound Cezanne-1 (see Figure S1C). Interestingly, even though residues 176–180, 224–225, and 367–372 are remote from either ubiquitin-binding site, their structural flexibilities are affected by substrate binding via so-far uncharacterized intramolecular communications.

### Different Binding Modes of Proximal and Distal Ubiquitin

**Figure 6:**
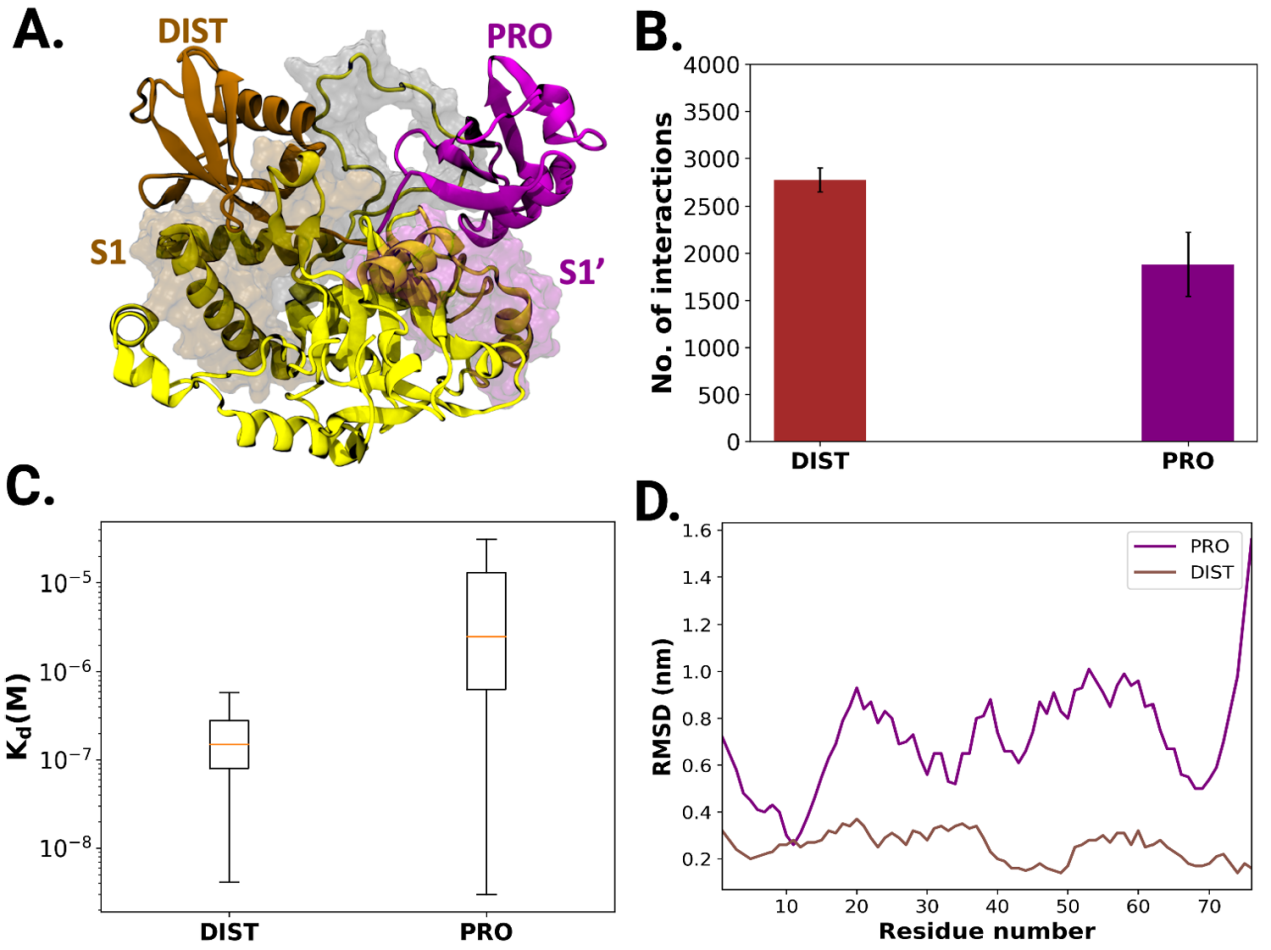
Proximal and distal ubiquitin exhibit different binding modes and affinities toward Cezanne-1. (A) Structural details of DIST and PRO ubiquitin binding to Cezanne-1 via their binding sites S1 and S1′ (PDB ID: 5LRV). (B) Number of protein–protein interactions between DIST and PRO ubiquitins and Cezanne-1 (cutoff 0.55 nm). (C) Box plot of predicted dissociation constants of DIST and PRO ubiquitins from Cezanne-1. K_d_ values were calculated from 3000 equidistant MD snapshots covering 3 μs. (D) Relative Cα RMSD of PRO (purple) and DIST (ochre) ubiquitin residues when *Cez^+/-^Ub*_2_ trajectories are aligned to the OTU domain.

Since differences in residual fluctuations can be seen for PRO and DIST ubiquitin-binding sites, their respective binding modes to Cezanne-1 were characterized. When aligning trajectories of the Cezanne-1 K11-linked diubiquitin complex to those of Cezanne-1, the relative RMSD (rRMSD) gives information about conformational transitions and differences in translational and rotational motions of the ubiquitins. The rRMSD of the distal ubiquitin is low (0.4 nm) in five independent replicates of 600 ns MD simulations. In contrast, the rRMSD of the proximal ubiquitin is high (reaches up to 3 nm) in all trajectories. Thus, the distal ubiquitin exhibits a stable binding, whereas the proximal ubiquitin undergoes significant structural dynamics (Figure S4). The less stable binding of proximal ubiquitin in comparison to distal ubiquitin is also confirmed by higher Cα rRMSD values of proximal ubiquitin residues than those of DIST ubiquitin residues (see Figure 6D).

The differences in dynamics between the two ubiquitin moieties (distal and proximal) can be rationalized by the number of intermolecular protein–protein interactions (Figure 6). Distal ubiquitin is involved in 2771 ± 128 interactions with Cezanne-1, whereas proximal ubiquitin engages in only 1884 ± 341 interactions. Both the number of interactions and their low standard error of means indicate a reproducible stabilization of the distal ubiquitin compared to the proximal one. The number and type of interactions (hydrophobic, electrostatic, etc.) plus residual desolvation energies contribute to the free energy of binding and thus the dissociation constant K_d_.^51,52^ PRODIGY allows the prediction of K_d_ based on an empirical model that includes interactions and surface effects. For distal ubiquitin, the model yields a median K_d_ of 2.5 μM and for proximal ubiquitin a significantly higher median K_d_ of 15 μM which is in agreement with the calculated differences in interprotein interactions and relative RMSD values. Upon hydrolysis of the Gly76-Lys11 iso-peptide bond between the two ubiquitin monomers, dissociation and release of the products must occur to complete the catalytic cycle. The above results suggest that, after cleavage of the iso-peptide bond, the dissociation of proximal ubiquitin from Cezanne-1 would occur first, followed by sequential release of distal ubiquitin. These results are in agreement with the experimental trap of only monoubiquitinylated Cezanne-1 in complex with the distal ubiquitin.

### Sequential Dissociation of Proximal Ubiquitin and Distal Ubiquitins from Cezanne-1

**Figure 7:**
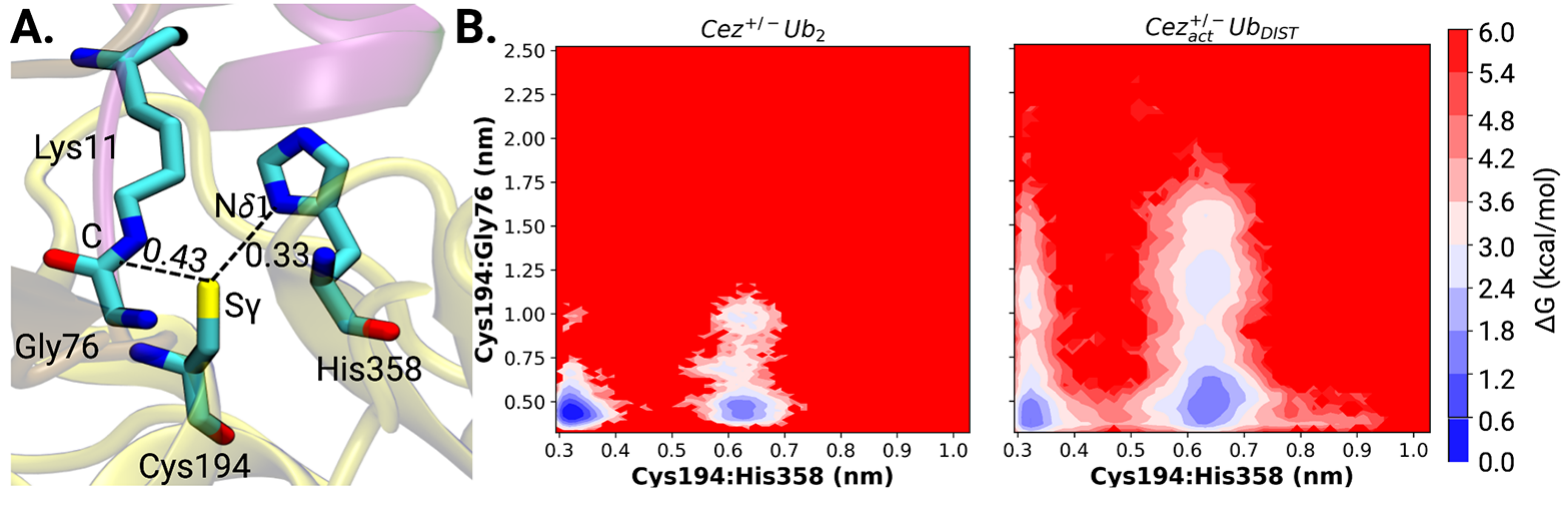
State of activation of Cezanne-1 in diubiquitinylated and monoubiquitinylated complexes. (A) Structural details of the active enzyme–substrate complex of *Cez*^+/-^*Ub*_2_. Relevant interatomic distances of the Cezanne-1 active site (Sγ-Cys194 and Nδ1-His358), and Sγ-Cys194 Cezanne-1 and the scissile bond between Gly76/Lys11 are labeled. The OTU domain of Cezanne-1 is given in yellow. PRO ubiquitin is in purple, and DIST is in ochre. (B) Free energy maps for the enzyme–substrate complex *Cez*^+/-^*Ub*_2_ (left) and the product state *Cez*^+/-^_*apo*_*Ub*_*DIST*_ (right) as a function of Cys194···His358 and Cys194···Gly76 distances. Dissociation of PRO ubiquitin favors the catalytic deactivation of Cezanne-1, which is followed by the removal of the C-terminal tail of DIST ubiquitin from the catalytic center.

During MD simulations of the ternary di-ubiquitin–protein complex, active states of Cezanne-1 (Figure 7A) are more frequently occurring than inactive states. In this pre-reactive state, the scissile bond approaches the catalytic Cys194 to within 0.30–0.43 nm, which is in agreement with QM/MM studies of the enzyme–substrate complex for other cysteine proteases.^63,64^ The short cysteinate distance to the protonated histidine (0.33 nm) is also found in QM/MM calculations. This shows that the MD simulations are able to provide a reliable picture of the enzyme–substrate (Michaelis) complex prior to peptide bond hydrolysis.

Differences in RMSFs, rRMSDs, number of protein–protein interactions, and calculated dissociation constants suggest that the proximal ubiquitin will dissociate first and give the DIST monoubiquitinylated Cezanne-1 complex.

In the substrate-bound state, both catalytically active, i.e., prereactive (Cys194···His358 distances of 0.30–0.36 nm and Cys194···Gly76 distances of 0.37–0.51 nm) and inactive (Cys194···His358 distances of 0.60–0.70 nm), states are present. Short catalytic site residue distances and short enzyme–substrate distances represent the active state of Cezanne-1 prior to the catalytic performance (with a probability of 0.77). The strong electrostatic interaction between the Cezanne-1 residue Glu157 and Lys33 of PRO ubiquitin is persistent and stabilizes the substrate positioning relative to the catalytic residues. This particular prereactive conformation favors a catalytically competent state of Cezanne-1. This indicates that Glu157 plays a key role in the catalytic activity albeit it may not directly be involved in the cleavage reaction mechanism. Similar effects are also observed in other OTU DUB proteases. For example, OTULIN uses a Cys/His/Asn and its bacterial equivalence RavD a Cys/His/Ser site to selectively recognize and position M1-linked diubiquitin, although only the first two residues perform the catalytic bond cleavage.^13,65^

After bond cleavage, in the product monoubiquitinylated *Cez*^+/-^_*act*_*Ub*_*DIST*_ state, two energy minima at Cys194···His358 distances of 0.30–0.34 nm/Cys194···Gly76 distances between 0.35 and 0.50 nm and 0.60–0.67 nm/0.37–0.62 nm can be seen. The minima are shallow, and conversion from the active (with a probability of occurrence of 0.26) to an inactive state (with a probability of 0.74) is facile even though the simulations started from an activated Cezanne-1. This indicates that, upon initial dissociation of PRO ubiquitin, the active site Cezanne-1 prefers a conversion back to a catalytically inactive state. The increase in Cys194···Gly76 distances also shows that the C-terminus of the DIST ubiquitin moves away from the active site of Cezanne-1. This information is not available from the mono-Ub Cezanne-1 crystal structure in which fixation of the distal ubiquitin leads to a short covalent Cys194-product bond and does not enable relaxation of the active site of Cezanne-1.

## Conclusion

Cezanne-1 OTU DUB undergoes a complex, multistage catalytic cycle when cleaving the iso-peptide bond between Gly76 of distal ubiquitin and Lys11 of proximal ubiquitin. Crystal structures of Cezanne-1 provide valuable insight into the K11-linkage selectivity and intermediates of the catalytic cycle. The use of ABPs and covalent stabilization of product states give initial ideas about structural changes during diubiquitin cleavage. However, they may not represent physiologically relevant states.

The full process of OTU activation, substrate recognition, cleavage, and product dissociation cannot be rationalized based on the static intermediate structures only. They may not give a complete picture of all accessible states. Whereas the static crystals structures of Mevissen et al.^18^ give valuable insight into intermediates during the catalytic cycle, MD simulations provide dynamic and atomistic insight into the catalytic cycle of Cezanne-1 by recovering substrate-free, substrate-bound, and product states of Cezanne-1 in physiological conditions. For example, the equilibrium between catalytically competent and incompetent states of Cezanne-1 in the absence of substrate or the deactivation of monoubiquitinylated Cezanne-1 cannot be obtained from protein crystal structures.

With MD simulations covering several microseconds, the conformational flexibility of Cezanne-1 during the activation and catalytic process becomes apparent. We show, for the first time, that the active site of Cezanne-1 dynamically shuttles between catalytically competent and incompetent states even in the absence of a substrate. Only the catalytically competent substrate-free Cezanne-1 becomes substrate accessible. Upon binding of the proximal ubiquitin, the prereactive conformation and active state are reached via strong electrostatic interactions between the Cezanne-1 residue Glu157 and Lys33 of PRO ubiquitin. Then, close positioning and proper orientation of the iso-peptide bond relative to cysteinate initiate the bond cleavage. The sequential release of first the loosely associated proximal ubiquitin induces the deactivation step of Cezanne-1 and the recovery of the ubiquitin-free state.

Cezanne-1 is a tentative therapeutic target since it is overexpressed in cancer cells.^29,30^ When targeting Cezanne-1 with novel therapeutics, the catalytic dyad, the ubiquitin-binding sites S1 and S1′, or maybe other allosteric sites can be addressed, in principle. The success of structure-guided drug design relies on the target structures to correspond to a physiological state. The currently available protein structures of Cezanne-1 are incorporating modifications in protein sequence, some unresolved loop regions, and covalent modifiers to stabilize the cysteine nucleophile in complex with the substrate and product. These lead to protein structures in nonphysiological states with different structural parameters, which need to be modified to recover physiologically relevant conformational states of Cezanne-1.

## Data Availability

OpenMM version 7.6 was used to perform MD simulations (freely available from https://openmm.org). GROMACS version 2022.2 packages (freely available from https://www.gromacs.org) were used for trajectory analysis. VMD version 1.9.4a55 is available from http://www.ks.uiuc.edu/Research/vmd/. Data visualization was carried out by using Seaborn version 3.5.1 (https://seaborn.pydata.org) and Matplotlib version 3.5.1 (https://matplotlib.org) libraries. The initial structures and openMM script are available from https://edmond.mpdl.mpg.de/privateurl.xhtml?token=59b4007d-62bc-414c-88c3-8df40a8b6a0c. All MD trajectories are stored on a local server and will be shared upon request due to the large file sizes.

## Supporting Information Available

PCA analysis of apo and holo Cezane-1, Cα RMSD per residue change in comparison to substrate-bound state, timeline evolution of Glu157···His358, Cys194···His358, Asn193···Leu294, Asn193···Cys194 distances in neutral and zwitterionic charge states of apo, RMSD profiles of proximal and distal ubiquitins (PDF)
