# Supplementary material for: Structural Dynamics of Lys11-Selective Deubiquitinylase Cezanne-1 during the Catalytic Cycle
Source: J Chem Inf Model. 2023 Mar 21;63(7):2084–94. doi: 10.1021/acs.jcim.2c01281 (PMC10091412; doi:10.1021/acs.jcim.2c01281)
Supplement: Supplementary file 1 — ci2c01281_si_001.pdf [file ci2c01281_si_001.pdf]

Supporting Information
Structural Dynamics of Lys11-Selective Deubiquitinylase Cezanne-1 during the Catalytic Cycle

Metehan Ilter,[†] Eric Schulze-Niemand,[†,‡] Michael Naumann,[‡] and Matthias Stein[*,†]

†*Molecular Simulations and Design Group, Max Planck Institute for Dynamics of Complex Technical Systems, 39106, Magdeburg, Germany*

‡*Medical Faculty, Institute for Experimental Internal Medicine, Otto von Guericke University, 39120, Magdeburg, Germany*

E-mail: matthias.stein@mpi-magdeburg.mpg.de

Formation of Catalytically Competent State of Cezanne-1 Destabilizes the Hydrogen Bonding between Asn193-Cys194

In the crystal structure of apo Cezanne-1, Asn193-Leu294 are at a short distance (0.30 nm) and there is a hydrogen bond interaction between them. In the neutral charge state Cez^{0}_{apo}, the short Asn193-Cys194 (dark blue) and Asn193-Leu294 (blue) interactions are persistent during the simulation (see **Figure S3B**, left).

Figure S3B (right) monitors the same inter-residue distances in $Cez^{+/-}_{apo}$. The loss of the hydrogen bonding between Cys194 and Asn193 leads to an increase in Asn193$\cdots$Leu294

distances. **Figure S3C** shows a final snapshot structure of an open substrate access channel.

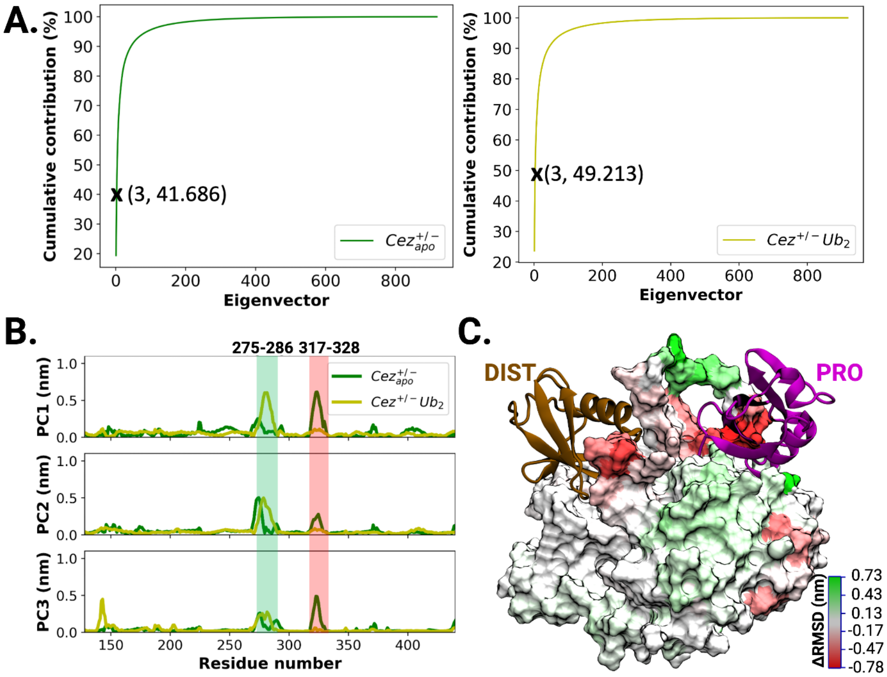

Figure S1: **A.** The contribution of eigenvectors to overall dynamics was obtained from the diagonalized covariance matrices of $Cez_{apo}^{+/-}$ (left) and $Cez^{+/-}Ub_2$ (right). The cumulative contribution of the first three eigenvectors to overall dynamics is denoted by a cross. **B.** Principal component analysis of $Cez_{apo}^{+/-}$ and $Cez^{+/-}Ub_2$. Cα fluctuation of residues projected along the first three eigenvectors, principal components (PCs). Residues with higher eigen RMSF values in comparison to $Cez^{+/-}Ub_2$ are shown in a green area corresponding to the V-loop, whereas residues with reduced eigen RMSF values are shown in a red area, DIST ubiquitin-binding site of $Cez^{+/-}Ub_2$. **C.** The difference in Cα RMSD per residue in comparison to physiologically reconstituted $Cez^{+/-}Ub_2$ trajectories. The OTU domain is shown in surface representation and colored according to changes in Cα RMSD per residue in comparison to $Cez^{+/-}Ub_2$. PRO and DIST ubiquitins are shown in the New Cartoon representation with purple and ochre colors, respectively. The green shaded areas represent the higher flexibility, which denotes, in particular, the V-loop and PRO ubiquitin-binding sites in comparison to the substrate-bound state. The red-shaded areas show the stabilized regions upon di-ubiquitin binding.

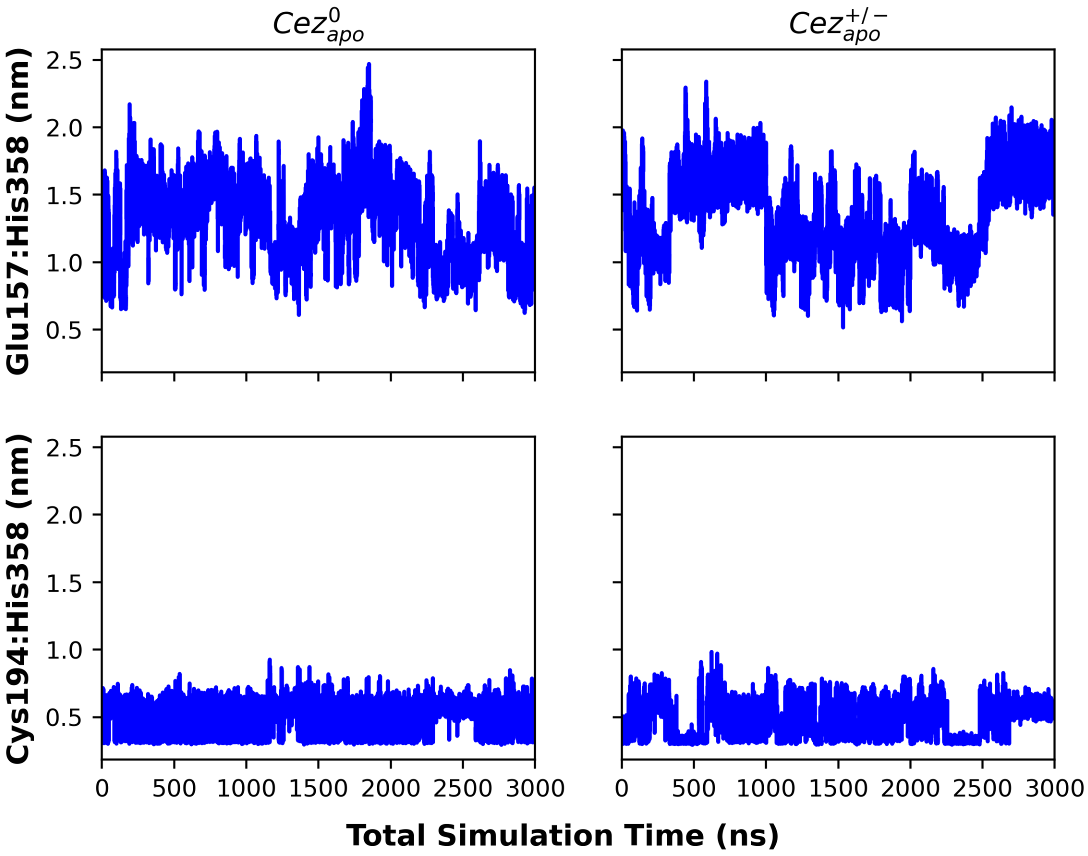

Figure S2: (**Top**) The timeline evolution of Glu157$\cdots$His358 distance and (**Bottom**) Cys194$\cdots$His358 distance in neutral (left) and charge-separate (right) states of substrate-free Cezanne-1. The timeline data is obtained by concatenating three independent trajectories of Cez^{0}_{apo} (left) and $Cez^{+/-}_{apo}$ (right).

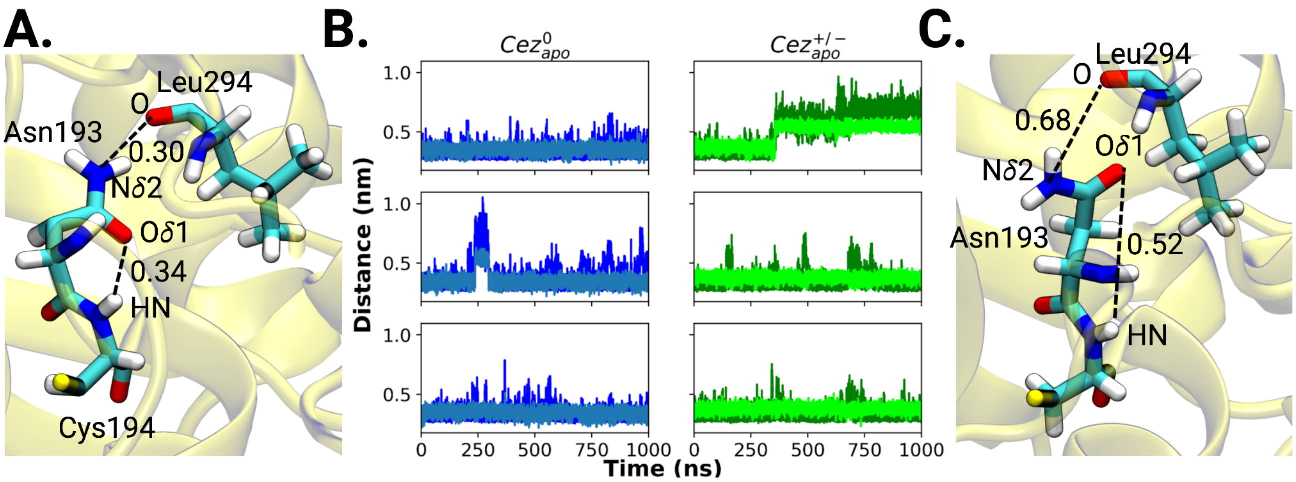

Figure S3: **A.** Initial structure of the substrate-free Cezanne-1. The access of DIST ubiquitin is obstructed by interactions between Asn193-Leu294 at 0.30 nm distance. In the catalytically non-competent state, hydrogen bonding between Oδ1-Asn193 and HN-Cys194 (Asn193$\cdots$Cys194) at 0.34 nm also brings Asn193 and Leu294 in close contact. **B.** Time evolution of Asn193$\cdots$Leu294 (dark blue and dark green) and Cys194$\cdots$Asn193 (light blue and light green) distances in neutral (left) and zwitterionic (right) charge states. It shows the prevalence of the close Asn193-Leu294 contact and the hydrogen bonding of Cys194-Asn193. As the hydrogen bonding between Cys194 and Asn193 is lost in the zwitterionic state, the Asn193$\cdots$Leu294 distance increases. **C.** A representative structure of $Cez^{+/-}_{apo}$, in which the loss of the Cys194-Asn193 hydrogen bond (0.52 nm) and the loss of Asn193-Leu294 interaction (0.68 nm) are shown.

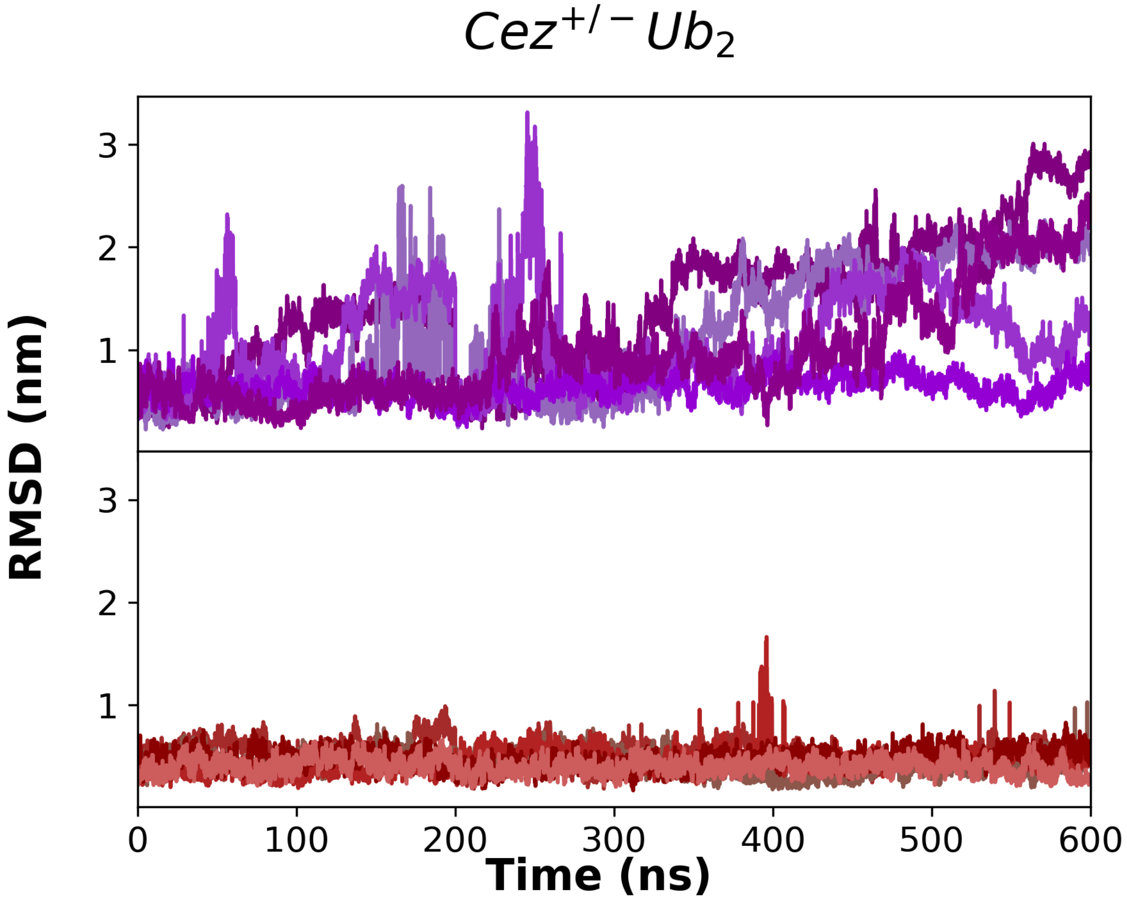

Figure S4: Backbone RMSD of ubiquitin relative to the OTU domain of crystallized Cezanne-1 (PDB ID: 5LRV). (**Top**) Five individual trajectories of 600 ns each of PRO (top) and (**Bottom**) DIST ubiquitins are shown.
